# Laparoscopic Cholecystectomy in a Child Using Slender Forceps

**DOI:** 10.21699/ajcr.v8i3.559

**Published:** 2017-05-01

**Authors:** Kazuaki Shibuya, Hideki Kawamura, Munenorix Tahara, Masahiro Takahashi, Akinobu Taketomi

**Affiliations:** 1 Department of Gastroenterological Surgery I Hokkaido University Graduate School of Medicine Kita 15, Nishi 7, Kita-ku, Sapporo 060-8638, Japan; 2 Department of Surgery, Hokkaido P.W.F.A.C Sapporo Kosei General Hospital Kita 3-Jo Higashi 8-chyome, Chuo-ku, Sapporo 060-0033, Japan

**Keywords:** Pediatric cholelithiasis, Laparoscopy, Needlescopy

## Abstract

Laparoscopic procedures in children are technically demanding because of reduced working space with careful monitoring of pneumoperitoneum pressure. We report a case of laparoscopic cholecystectomy performed in a 9-year-old boy using slender forceps which addressed all the above mentioned concerns. This shows a possibility of needlescopic surgery in children.

## INTRODUCTION

Cholelithiasis in children is not an uncommon condition. Laparoscopic cholecystectomy is a preferred mode of treatment. Laparoscopy in children is challenging as surgeons have to work in a restricted space with low pressure pneumoperitoneum [1]. Single port laparoscopy using slender forceps is increasingly practiced nowadays [2]. In pediatric laparoscopic surgery, slender forceps are useful in performing appendectomy, colectomy, and inguinal hernia repair [3]. Herein we describe surgical technique of laparoscopic cholecystectomy using slender forceps.

## CASE REPORT

A 9-year-old boy (height:131.3 cm, weight: 22.7 kg) with the diagnosis of cholelithiasis was booked for laparoscopic cholecystectomy. After preoperative optimization, a small abdominal incision for insertion of the camera port (5-mm) was made in umbilicus. The pressure of pneumoperitoneum was kept at 6 mmHg, which is lower than that for an adult (8-10 mmHg). To use ultrasonically activated device (USAD), a 5-mm port was inserted below the xiphoid process, but to reduce the interference of the forceps within the restricted working space and for aesthetic considerations, two 2-mm diameter grasper forceps (BJ needle; NITI-ON, Chiba, Japan) were inserted in the right hypochondrium for the surgeon’s left hand and the assistant. Cystic artery was coagulated with USAD. Cystic duct was double ligated using 4-0 absorbable sutures. Duration of surgery was 45 min, and there was minimal blood loss.

A 9-mm black stone was found within the gallbladder. Calculus analysis indicated that its composition was 36% calcium carbonate, 33% calcium phosphate, and 31% calcium bilirubinate. The patient was discharged on the fourth day after surgery without any complication.

## DISCUSSION

Laparoscopic cholecystectomy in children requires several adjustments in technique. In the present case, because of the restricted space we used slender forceps. We practice needlescopic surgery for the colon related operations as well as single port laparoscopic cholecystectomy at our institute. We applied our knowledge and skill for needlescopic cholecystectomy for the first time in our hospital. In our case, abdominal pressure was maintained at 6 mmHg and no effect on respiratory dynamics or hemodynamics was observed, and the surgery could be performed while maintaining a satisfactory operative field. In conclusion, slender forceps can be used to perform cholecystectomy safely thus promoting needlescopic surgery which further have benefits of barely visible scars and negligible postoperative pain compared to conventional laparoscopy.

## Footnotes

**Source of Support:** Nil

**Conflict of Interest:** None declared

